# Parameters of State in the Global Thermodynamics of Binary Ideal Gas Mixtures in a Stationary Heat Flow

**DOI:** 10.3390/e25111505

**Published:** 2023-10-31

**Authors:** Anna Maciołek, Robert Hołyst, Karol Makuch, Konrad Giżyński, Paweł J. Żuk

**Affiliations:** 1 Institute of Physical Chemistry, Polish Academy of Sciences, Kasprzaka 44/52, 01-224 Warszawa, Poland; kmakuch@ichf.edu.pl (K.M.); kgizynski@ichf.edu.pl (K.G.);; 2 Max-Planck-Institut für Intelligente Systeme Stuttgart, Heisenbergstr. 3, D-70569 Stuttgart, Germany; 3 Department of Physics, Lancaster University, Lancaster LA1 4YB, UK

**Keywords:** non-equilibrium thermodynamics, entropy and internal energy, mixtures and parameters of state

## Abstract

In this paper, we formulate the first law of global thermodynamics for stationary states of the binary ideal gas mixture subjected to heat flow. We map the non-uniform system onto the uniform one and show that the internal energy $U(S^{*},V,N_{1},N_{2},f_{1}^{*},f_{2}^{*})$ is the function of the following parameters of state: a non-equilibrium entropy $S^{*}$, volume *V*, number of particles of the first component, $N_{1}$, number of particles of the second component $N_{2}$ and the renormalized degrees of freedom. The parameters $f_{1}^{*},f_{2}^{*}$, $N_{1},N_{2}$ satisfy the relation $\left(N_{1}/\left(N_{1}+N_{2}\right)\right)f_{1}^{*}/f_{1}+\left(N_{2}/\left(N_{1}+N_{2}\right)\right)f_{2}^{*}/f_{2}=1$ ($f_{1}$ and $f_{2}$ are the degrees of freedom for each component respectively). Thus, only 5 parameters of state describe the non-equilibrium state of the binary mixture in the heat flow. We calculate the non-equilibrium entropy $S^{*}$ and new thermodynamic parameters of state $f_{1}^{*},f_{2}^{*}$ explicitly. The latter are responsible for heat generation due to the concentration gradients. The theory reduces to equilibrium thermodynamics, when the heat flux goes to zero. As in equilibrium thermodynamics, the steady-state fundamental equation also leads to the thermodynamic Maxwell relations for measurable steady-state properties.

## 1. Introduction

In classical thermodynamics, the internal energy, U(S,V,N), of a one-component ideal gas is a function of three parameters: entropy, *S*, volume *V* and the number of particles *N*. Each parameter of state represents one independent way of system’s energy exchange with the external world. For fixed *N*, there are two ways of energy change: heat and work. The infinitesimal change of the internal energy satisfies the equation dU=TdS−pdV, where the first term is the heat and the last is the work term. Until recently, no such description was available for systems in non-equilibrium states, subjected to energy flow.

The classical theory of irreversible (non-equilibrium) thermodynamics [1] is based on three differential non-linear equations representing conservation of mass, momentum (Navier–Stokes equation) and energy. These conservation laws are supplemented by the assumption of the local equilibrium, corresponding local equations of state and constitutive relations between fluxes and thermodynamic forces. The solutions of these equations are given in terms of velocity, v(r,t), temperature, T(r,t) and number density of particles, n(r,t) profiles. In the stationary states, the profiles and fluxes depend only on the position in space r, not on time, *t*. The assumption of the local equilibrium is crucial in the formulation of the local equations of state. For the ideal gas it is justified, but contested for interacting systems [2]. The assumption of local equilibrium for the ideal gas is valid for a small temperature gradient, i.e., such that $l_{fp}\left|\nabla T\right|/T\ll 1,$ for the mean free path of the molecules, $l_{fp}$ [3]. At the pressure of 1 bar at room temperature, the mean free path is of the order of $l_{fp}\approx$ 100 nm. In such conditions, the assumption of local equilibrium breaks down only for temperature gradients higher than $10^{7}$ K/cm. Thus, this assumption is even satisfied inside the Sun! This local description contains the first law of thermodynamics, but in a different form than that given in the equilibrium thermodynamics and based on a few *global* parameters of state. In our recent paper [4] we provided the latter, i.e., global thermodynamic description for the ideal gas in a heat flow.

We have previously [4] studied the one-component ideal gas in a heat flow between two parallel walls at distance *L*, kept at two different temperatures $T_{1}\left(z=0\right)>T_{2}\left(z=L\right)$. The local equilibrium gives the local pressure, p(z), and the internal energy per unit volume, u(z):

(1) $$p\left(z\right)=k_{B}T\left(z\right)n\left(z\right),$$

and

(2) $$u\left(z\right)=\frac{3}{2}n\left(z\right)k_{B}T\left(z\right),$$

with Boltzmann constant $k_{B}$, particle number density $n\left(z\right)$, and the temperature $T\left(z\right)$ at position *z*. We have shown rigorously that when we integrate both equations over the volume of the system we can formulate the global thermodynamics with the internal energy as a function of a few parameters of state. After integration we obtain:

(3) $$pV=Nk_{B}\frac{T_{2}-T_{1}}{\log\frac{T_{2}}{T_{1}}}.$$

and

(4) $$U=\frac{3}{2V}Nk_{B}\frac{T_{2}-T_{1}}{\log\frac{T_{2}}{T_{1}}}.$$

We identify the system’s temperature, $T^{*}$,

(5) $$T^{*}=\frac{T_{2}-T_{1}}{\log\frac{T_{2}}{T_{1}}}.$$

In this way we made a mapping of the non-uniform system into the uniform one. We observe that obtained equations have the same form as in equilibrium for the ideal gas at temperature $T^{*}$. We demanded such form because, after the mapping, we treat the system as the uniform one as in equilibrium. Moreover, the obtained equations of state must reduce to the equilibrium equations of state when the heat flux is zero. Now we define the internal energy as a function of three parameters of state $U(S^{*},V,N)$, with the thermodynamic relation:

(6) $$\begin{array}{cc} {\left(\frac{\partial S^{*}}{\partial U}\right)}_{V,N} & =\frac{1}{T^{*}},\\ {\left(\frac{\partial S^{*}}{\partial V}\right)}_{U,N} & =\frac{p}{T^{*}}. \end{array}$$

This mapping gives us the same formal structure as we know from equilibrium. The entropy $S^{*}$ is responsible for the **net heat** that enters or leaves the system [5,6,7] and changes the internal energy. In general, the heat flows through the system all the time without changing the internal energy. Upon any process we would like to know how much of the heat transferred to the system changes the internal energy. This heat is called net heat and is given in the differential form by $T^{*}dS^{*}$.

$S^{*}$ is only part of the total entropy of the system. The total entropy of the system, $S_{tot}=A\int dzs\left(z\right)$, where s(z) is the volumetric entropy density given by local equilibrium assumption within irreversible thermodynamics, can be written as a sum [4]:

(7) $$\begin{array}{cc} S_{\mathrm{tot}}\left(U,V,\frac{T_{2}}{T_{1}}\right) & =S^{*}\left(U,V\right)+\Delta S\left(U,V,\frac{T_{2}}{T_{1}}\right),\\ \Delta S\left(U,V,T_{2}/T_{1}\right) & =Nk_{B}\log\left[{\left(\frac{T_{2}}{T_{1}}\right)}^{5/4}{\left(\frac{\log\frac{T_{2}}{T_{1}}}{\frac{T_{2}}{T_{1}}-1}\right)}^{5/2}\right]. \end{array}$$

Only $S^{*}$ governs net heat in the system (heat absorbed/released in the system). ΔS controls the dissipative background and solely depends on the entropy production given by [1] $\sigma =-A\int_{0}^{L}dz\;\kappa \nabla T\left(z\right)\cdot \nabla \frac{1}{T\left(z\right)}=\frac{A\kappa }{L}\left(\frac{T_{2}}{T_{1}}+\frac{T_{1}}{T_{2}}-2\right)$, where κ is the heat conductivity. The difference between the total entropy and $S^{*}$ vanishes, $\Delta S\left(U,A,L,T_{2}/T_{1}\right)\to 0$, when the system approaches the equilibrium state, $T_{2}/T_{1}\to 1$. Therefore, $S^{*}$ becomes in this limit the equilibrium entropy. Nonetheless the formal dependence of $S^{*}$ on *U* and *V* at non-equilibrium state in a heat flow is the same as at equilibrium.

In this paper we want to apply the same mapping procedure to the binary mixture of ideal gases. The big difference between previous work and the current one is the fact that apart from the number density profile and the temperature profile we have additional profiles of the number densities of each component. As we shall see, these additional profiles lead to new parameters of state in the non-equilibrium state, which have no direct counterpart at equilibrium. The purpose of this work is to formulate the first law of global thermodynamics for ideal gas binary mixture in the heat flow. The paper is organized as follows: In Section 2, we recall the equilibrium properties of the ideal gas mixture. In Section 3 we discuss this mixture enclosed between two parallel walls at different temperatures and solve the equations of irreversible thermodynamics. Section 4 and Section 5 contain the main results of the present study. In Section 4, we define all parameters of state for the mixture and perform the mapping of non-uniform system into the uniform one. We introduce the first law of non-equilibrium thermodynamics, which follows from these parameters, and discuss some of its consequences in Section 5. We discuss the results in Section 6.

## 2. Preliminaries

We consider a binary mixture of ideal gases enclosed between two parallel walls separated from each other by a distance *L* in the *z* direction. The volume of a system is V=AL, where *A* is an area in the x−y plane. The components of the mixture have number densities $n_{1}$ and $n_{2}$, such that $n=N/V=n_{1}+n_{2}$ is the total density. The equation of state of ideal gas at pressure *p* and temperature *T*, $p=nk_{B}T$, where $k_{B}$ is the Boltzmann constant, can be written as a sum of partial pressures $p_{i}$

(8) $$p=p_{1}+p_{2}=n_{1}k_{B}T+n_{2}k_{B}T$$

which is the Dalton’s law [8].

The internal energy density (per volume) is the sum of the internal energy density $u_{i}$ (i=1,2) of the two components separately considered $u=u_{1}+u_{2}$. From the classical equipartition theorem applied to ideal gas it follows that

(9) $$u_{i}=\frac{f_{i}}{2}n_{i}k_{B}T=\frac{f_{i}}{2}p_{i},\;\;i=1,2$$

where $f_{i},i=1,2$ are translational and rotational degrees of freedom of the *i* component. $f_{i}$ takes the value 3 for monoatomic, 5 for diatomic, and 6 for polyatomic gas. Often, the ideal gas law (9) is written using dimensionless specific heat capacity at constant volume $c_{v}^{\left(i\right)}=\frac{f_{i}}{2}$.

The equilibrium entropy density of a binary mixture is

(10) $$n\cdot s=ns^{\left(0\right)}+k_{B}\left[n\ln n-n_{1}\ln n_{1}-n_{2}\ln n_{2}\right].$$

Here, $ns^{\left(0\right)}=n_{1}s_{1}^{\left(0\right)}+n_{2}s_{2}^{\left(0\right)}$, where $s_{i}^{\left(0\right)}\;\;\left(i=1,2\right)$ is the entropy per particle of the two components separately considered. The second term in (10) is the mean-field expression for the mixing entropy. For ideal gas, one has

(11) $$\frac{s_{i}^{\left(0\right)}}{k_{B}}=\frac{f_{i}}{2}+1+\frac{f_{i}}{2}\ln\left(\frac{2\Phi_{i}u_{i}}{f_{i}k_{B}n_{i}^{1+2/f_{i}}}\right),$$

where $\Phi_{i}$ is independent of the thermodynamic state of the gas and has dimension of $n^{2/f_{i}}/T$. For a monoatomic ideal gas, a quantum mechanical theory of Sacur–Tetrode predicts that the constant $\Phi_{i}$ depends only upon the mass of the gas particle [9,10].

The total energy and entropy are given by

(12) $$U=\int_{V}ud^{3}r\;\mathrm{and}\;S=\int_{V}nsd^{3}r,$$

which reduces to U=Vu and S=Ns for homogeneous systems. In the absence of external fields, the equilibrium ideal gas is homogeneous.

From fundamental thermodynamic relation in terms of entropy, the differential dS has the following form

(13) $$dS=\frac{1}{T}dU+\frac{p}{T}dV-\frac{\mu_{1}}{T}dN_{1}-\frac{\mu_{2}}{T}dN_{2}.$$

In the next section we will use the chemical potential difference defined as $\frac{\mu }{T}=-{\left(\frac{\partial S}{\partial \varphi }\right)}_{u,n}$, where $\varphi =n_{1}-n_{2}$. It is given by

(14) $$\frac{\mu }{T}=\frac{\mu_{1}^{\left(0\right)}-\mu_{2}^{\left(0\right)}}{2T}+\frac{k_{B}}{2}\ln\frac{x_{1}}{1-x_{1}},$$

where $x_{i}=n_{i}/n$ is the number fraction of *i* component; $x_{1}+x_{2}=1$ in the absence of chemical reactions. $\frac{\mu_{i}^{\left(0\right)}}{T}=-{\left(\frac{\partial n_{i}s_{i}^{\left(0\right)}}{\partial n_{i}}\right)}_{u_{i},n_{j\neq i}}$ is the chemical potential of the component *i* individually considered; it depends only on temperature *T* and pressure *p*.

## 3. Ideal Gas Mixture in Heat Flow

Now, we introduce heat flow into the system by setting different temperatures on the walls, i.e.,

(15) $$\begin{array}{cc} T\left(z=0\right) & =T_{1},\\ T\left(z=L\right) & =T_{2}. \end{array}$$

In the non-equilibrium state induced by this boundary condition, the system becomes inhomogeneous and one has to consider spatially varying temperature $T\left(r\right)$, pressure $p\left(r\right)$, density $n\left(r\right)$ and difference in number densities $\varphi \left(r\right)$. In the hydrodynamic limit, the time evolution of the binary mixture is given by the conservation laws for *n*, φ, momentum and energy supplemented by the assumption of local equilibrium, relations between thermodynamic forces and fluxes, and thermodynamic equations of state [1]. Note that ideal gas satisfies the local equilibrium exactly. This fact describes the strict absence of spatial correlations between particles, which is, of course, not the case for non-ideal systems.

We focus here on a stationary state with vanishing gas velocity field and constant pressure across the system. The latter, together with $n\left(r\right)$ and $\varphi \left(r\right)$, follows from conservation of mass and momentum. The assumptions v=0 and $p\left(r\right)=p$ are in agreement with our previous simulation studies [11,12,13] (for gas–liquid evaporating systems [11,12] and for Lennard–Jones fluid volumetrically heated [13]), which show that mechanical equilibrium is established very fast (in comparison to heat flow). Thus, the balance equations for φ and energy simplify to

(16) $$\begin{array}{ccc} \frac{\partial \varphi }{\partial t} & = & -2\partial_{i}J_{i}^{d},\\ \frac{\partial e}{\partial t} & = & -\partial_{i}J_{i}^{Q}, \end{array}$$

where $J^{d}\equiv J^{d,1}=-J^{d,2}$ is the diffusion current and $J^{q}$ is the heat current. We choose the following phenomenological expressions for $J^{d}$ and $J^{q}$ [1]:

(17) $$\begin{array}{ccc} J^{d} & = & -L_{11}\nabla \left(\frac{\mu }{T}\right)+L_{12}\nabla \left(\frac{1}{T}\right),\\ J^{q} & = & -L_{21}\nabla \left(\frac{\mu }{T}\right)+L_{22}\nabla \left(\frac{1}{T}\right) \end{array}$$

In steady state, $J^{d}=0$ because the system has no contact with particles reservoir.

First, we ignore the thermodiffusion effect, i.e., we assume that $L_{12}=0$. We also neglect the other cross-term by setting $L_{21}=0$. As a result, Equation (17) reduces to

(18) $$\nabla \left(\frac{\mu }{T}\right)=0$$

and

(19) $$\nabla T=const\Rightarrow \nabla^{2}T=0.$$

The latter yields a linear temperature profile (the system is translationally invariant in x,y directions)

(20) $$T\left(z\right)=T_{1}+\left(T_{2}-T_{1}\right)\frac{z}{L}.$$

From Equation (18) we determine the relationship between the local number fraction $x_{1}\left(z\right)$ of the component 1 and the temperature. The local equilibrium allows us to use the local form of Equation (14) for μ/T; it depends on $T\left(z\right),x_{1}\left(z\right),x_{2}\left(z\right)$, and the pressure *p*, which is constant throughout the system. Introducing $\tilde{T}\left(z\right)=T\left(z\right)/T_{1}$, Equation (18) with the use of Equation (14) gives

(21) $$\nabla \ln\frac{x_{1}\left(z\right)}{x_{2}\left(z\right)}=\nabla \ln\tilde{T}{\left(z\right)}^{\frac{f_{1}-f_{2}}{2}}.$$

With $x_{1}\left(z\right)=1-x_{2}\left(z\right)$ we find

(22) $$x_{1}\left(z\right)=\frac{N_{1}\tilde{T}{\left(z\right)}^{(f_{1}-f_{2})/2}}{N_{2}+N_{1}\tilde{T}{\left(z\right)}^{(f_{1}-f_{2})/2}}.$$

Note that if $f_{1}=f_{2}$, temperature inhomogeneity does not induce spatial variation of the composition, unless we take into account the effect of thermodiffusion, i.e., $L_{12}\neq 0$. In that case we find generally

(23) $$\nabla \ln\frac{x_{1}\left(z\right)}{x_{2}\left(z\right)}=\left[\frac{f_{1}-f_{2}}{2}-\frac{2L_{12}}{k_{B}L_{11}T\left(z\right)}\right]\nabla \ln\tilde{T}\left(z\right).$$

Onsager coefficients $L_{12}$ and $L_{11}$ are assumed to be independent on the concentration and temperature [14], therefore the solution of Equation (23) is

(24) $$x_{1}\left(z\right)=\frac{N_{1}\tilde{T}{\left(z\right)}^{(f_{1}-f_{2})/2}}{N_{2}e^{\frac{2L_{12}}{k_{B}T_{1}L_{11}}\left(1-\frac{1}{\tilde{T}\left(z\right)}\right)}+N_{1}\tilde{T}{\left(z\right)}^{(f_{2}-f_{2})/2}}.$$

**Figure 1:**
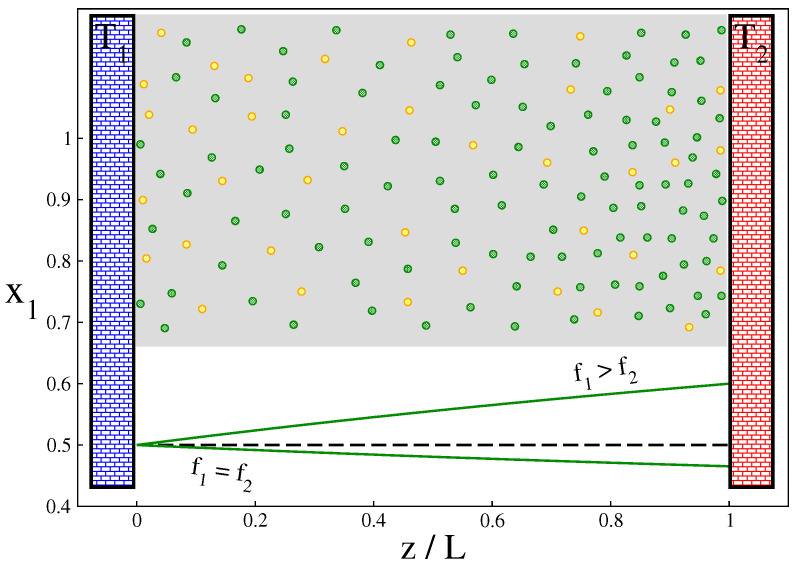
Schematic illustration of a binary ideal gas mixture between two parallel walls held at different temperatures $T_{2}$ and $T_{1}$. The resulting temperature gradient induces a gradient of fractions $x_{i}$ of the mixture components. Solid (green) lines indicate profiles of $x_{1}\left(z/L\right)$. Two cases are shown: (i) $f_{1}>f_{2}$ for which the profile is given by Equation (22) and (2) $f_{1}=f_{2}$ for which the profile is given by Equation (A3). For the latter case, we took $\alpha_{12}=0.3432$, which corresponds to the Ne-He pair at $T_{1}=300$ K. In both cases the temperature profile is given by (20) with the reduced temperature gradient $r=\left(T_{2}-T_{1}\right)/T_{1}$ chosen to be 0.5 and $N_{1}=N_{2}$.

This solution clearly shows that in the non-equilibrium steady state the difference $f_{1}-f_{2}$ is a relevant parameter. An analogy can be drawn here with the order parameter: for non-zero $f_{1}-f_{2}$, the profile $x_{1}\left(z\right)$ is *qualitatively* different from that for the zero difference $f_{1}-f_{2}$. Specifically, for $f_{1}-f_{2}>0$, the concentration of the first component of the mixture (with more degrees of freedom) increases towards the hotter wall, while for $f_{1}=f_{2}$ the opposite is true (see Figure 1). This observation is important in identifying the steady-state parameters in constructing global thermodynamics for this system. (In Appendix B, we mention other approaches to thermodiffusion involving different, measurable thermodiffusion coefficients and relate them to the Onsager ones.)

The density profile follows from the local equation of state and the condition of constant pressure at steady state, $n\left(z\right)=p/k_{B}T\left(z\right)$. For a given number of particles, $N=A\int_{0}^{L}dz\;n\left(z\right)$, this determines pressure as

(25) $$p=\frac{N}{V}k_{B}\frac{T_{2}-T_{1}}{\log\frac{T_{2}}{T_{1}}}.$$

Finally, the total energy of a mixture is the sum of the energies of the both components of the mixture (see Equations (9) and (12))

(26) $$U=U_{1}+U_{2}=A\frac{f_{1}}{2}k_{B}\int_{0}^{L}n\left(z\right)x_{1}\left(z\right)T\left(z\right)dz+A\frac{f_{2}}{2}k_{B}\int_{0}^{L}n\left(z\right)x_{2}\left(z\right)T\left(z\right)dz.$$

Thus, the set of independent parameters controlling the stationary state is $T_{1}$, $T_{2}$, *A*, *L*, *N*, $N_{1}$, $f_{1}$, $f_{2}$.

## 4. Non-Equilibrium Parameters and Functions of State

In this section we start constructing non-equilibrium thermodynamics. To this end we rewrite the total energy (per volume) as follows

(27) $$\begin{array}{c} u=\frac{U}{V}=\frac{f_{1}^{*}}{2}n_{1}k_{B}T^{*}+\frac{f_{2}^{*}}{2}n_{2}k_{B}T^{*}, \end{array}$$

where $n_{i}=N_{i}/V=\left(1/L\right)\int_{0}^{L}n_{i}\left(z\right)dz$. This has the same form as in equilibrium but with temperature *T* replaced by $T^{*}$ and $f_{1},f_{2}$ replaced by parameters $f_{1}^{*},f_{2}^{*}$, which we call the “effective” degrees of freedom. From Equation (26), it follows that

(28) $$f_{1}^{*}=f_{1}\frac{\frac{1}{L}\int_{0}^{L}n\left(z\right)x_{1}\left(z\right)\frac{k_{B}T\left(z\right)}{2}dz}{n_{1}\frac{k_{B}T^{*}}{2}}=f_{1}p\frac{\frac{1}{L}\int_{0}^{L}x_{1}\left(z\right)dz}{n_{1}k_{B}T^{*}}$$

and

(29) $$f_{2}^{*}=f_{2}\frac{\frac{1}{L}\int_{0}^{L}n\left(z\right)x_{2}\left(z\right)\frac{k_{B}T\left(z\right)}{2}dz}{n_{2}\frac{k_{B}T^{*}}{2}}=f_{2}p\frac{\frac{1}{L}\int_{0}^{L}x_{2}\left(z\right)dz}{n_{2}k_{B}T^{*}}$$

where we have used $n\left(z\right)T\left(z\right)=p/k_{B}=const$. The parameter $T^{*}$ can be determined from the requirement that the pressure

(30) $$p=k_{B}\frac{1}{L}\int_{0}^{L}\left(n_{1}\left(z\right)T\left(z\right)+n_{2}\left(z\right)T\left(z\right)\right)dz=p_{1}+p_{2}.$$

Then, Equation (30) can be written as

(31) $$p=n_{1}k_{B}T^{*}+n_{2}k_{B}T^{*},$$

which is to say that partial pressures in steady state are related to the non-equilibrium energies in the same way as in equilibrium (see Equation (9)) but with $f_{i}$ replaced by $f_{i}^{*}$. Solving Equation (31) with the use of Equation (25), we find

(32) $$T^{*}=\frac{p}{k_{B}}\frac{1}{n_{1}+n_{2}}=\frac{p}{k_{B}}\frac{V}{N}=\frac{T_{2}-T_{1}}{\ln\frac{T_{2}}{T_{1}}}$$

$T^{*}$ can be interpreted as an “average” temperature in the non-equilibrium steady state. Eliminating *p* in expressions for $f_{1}^{*},f_{2}^{*}$ we find:

(33) $$\begin{array}{cc} \; & f_{1}^{*}=\frac{f_{1}}{x_{1}}\frac{1}{L}\int_{0}^{L}x_{1}\left(z\right)dz,\\ \; & f_{2}^{*}=\frac{f_{2}}{x_{2}}\frac{1}{L}\int_{0}^{L}x_{2}\left(z\right)dz. \end{array}$$

Using $x_{2}\left(z\right)=1-x_{1}\left(z\right)$ we can see that the new parameters of state $f_{1}^{*},f_{2}^{*}$ are not independent but obey the following relation:

(34) $$\frac{f_{1}^{*}}{f_{1}}x_{1}+\frac{f_{2}^{*}}{f_{2}}x_{2}=1.$$

We note that even if both components of the mixture have the same number of degrees of freedom $f_{1}=f_{2}$, the effective parameters $f_{1}^{*},$ are not equal in non-equilibrium steady states providing we do not neglect thermal diffusion (see Equation (24)). Using Equations (20) and (22) for the profiles of the number fraction $x_{1}\left(z\right)$ of the component 1 and temperature T(z), we can express the steady-state variable $f_{1}^{*}$ in terms of the control parameters. The explicit formulas can be obtained for the case of $f_{1}\neq f_{2}$ in terms of the special functions as presented in Appendix A. For small, reduced temperature gradients $r=\left(T_{2}-T_{1}\right)/T_{1}$ and for $N_{1}=N_{2}$ we find

(35) $$f_{1}^{*}\approx \frac{f_{1}}{x_{1}}\left(\frac{1}{2}+\frac{f_{1}-f_{2}}{16}r\right)+O\left(r^{2}\right).$$

If $N_{1}\neq N_{2}$, the expression for $f_{1}^{*}$ in this limit is more complicated, but has a similar structure in terms of dependence on *r*. The coefficients are functions of $N_{1}/N_{2}$ and $f_{1},f_{2}$ but not of $T_{1}$ as shown in Appendix A. In the case of $f_{1}=f_{2}$, for which $x_{1}\left(z\right)$ given by Equation (24), the integral cannot be expressed in a closed form.

In the next step in the construction of global thermodynamics, we note that because the Equations (27) and (31) have the same structure as the equilibrium equation of state, we may formally write:

(36) $$\begin{array}{c} S^{*}={\left(S_{1}^{*}\right)}^{\left(0\right)}+{\left(S_{2}^{*}\right)}^{\left(0\right)}-N_{1}\ln\frac{N_{1}}{N}-N_{2}\ln\frac{N_{2}}{N}, \end{array}$$

with

(37) $$\begin{array}{c} \frac{{\left(S_{i}^{*}\right)}^{\left(0\right)}}{N_{i}k_{B}}=\frac{f_{i}^{*}}{2}+1+\frac{f_{i}^{*}}{2}\ln\left[\frac{2\Phi_{i}U}{k_{B}\left(N_{1}f_{1}^{*}+N_{2}f_{2}^{*}\right)}{\left(\frac{V}{N_{i}}\right)}^{2/f_{i}^{*}}\right]. \end{array}$$

Equation (36) has the functional form of the equilibrium fundamental relation for a binary ideal gas mixture with $f_{i}$ replaced by $f_{i}^{*}$ (compare Equations (10) and (11)). We will treat Equation (36) as a definition of the *non-equilibrium* steady-state entropy $S^{*}\left(U,V,N_{1},N_{2},f_{1}^{*},f_{2}^{*}\right)$ that also provides the *fundamental relation for the non-equilibrium steady state*. We note that $S^{*}$ differs from total entropy defined as $S_{tot}=A\int dzs\left(z\right)$, where s(z) is the volumetric entropy density given by local equilibrium assumption within irreversible thermodynamics. In comparison with the equilibrium entropy, $S^{*}$ depends on additional two state parameters $f_{1}^{*}$ and $f_{2}^{*}$. As a consequence, for the difference $dS^{*}$ we find:

(38) $$\begin{array}{c} dS^{*}=\frac{dU}{T^{*}}+\frac{p}{T^{*}}dV-\frac{\mu_{1}^{*}}{T^{*}}dN_{1}-\frac{\mu_{2}^{*}}{T^{*}}dN_{2}-\frac{F_{1}}{T^{*}}df_{1}^{*}-\frac{F_{2}}{T^{*}}df_{2}^{*}, \end{array}$$

where

(39) $$\begin{array}{cc} \frac{\mu_{i}^{*}}{k_{B}T^{*}} & =-{\left(\frac{\partial S^{*}}{\partial N_{i}}\right)}_{U,V,N_{j\neq i},f_{1}^{*},f_{2}^{*}}\\ \; & =-\frac{f_{i}^{*}}{2}\ln\frac{\Phi_{i}k_{B}T^{*}}{n_{i}^{2/f_{1}^{*}}}+\ln x_{i} \end{array}$$

and

(40) $$\frac{F_{i}}{T^{*}}=-{\left(\frac{\partial S^{*}}{\partial f_{i}^{*}}\right)}_{U,V,N_{1},N_{2},f_{j\neq i}^{*}}=-\frac{N_{i}}{f_{i}^{*}}\left(\ln x_{i}-\frac{\mu_{i}^{*}}{T^{*}}\right).$$

Equations (32)and (35) provide the effective parameters of state, $T^{*}$ and $f_{1}^{*}$ for given control parameters $T_{1},T_{2}$ and $N_{1},N_{2}$ at fixed $N_{1}+N_{2}$.

## 5. First Law and Its Consequences

We now consider the change of the total internal energy dU in our system. From Equation (38), we have

(41) $$\begin{array}{c} dU=T^{*}dS^{*}-pdV+\mu_{1}^{*}dN_{1}+\mu_{2}^{*}dN_{2}+F_{1}df_{1}^{*}+F_{2}df_{2}^{*}. \end{array}$$

As argued in Ref. [4], the change in internal energy dU in the very slow process of moving from one steady state to another by varying the control parameters (so that the pressure remains homogeneous) is given by

(42) dU=đQ+đW,

where đQ is the net heat entering the system during this transition, and for the *fixed* number of mixture components the mechanical work done is

(43) đW=−pdV.

The Equation (42) is therefore the first law of thermodynamics for non-equilibrium steady states where the net heat identified by Equations (42) and (38) (at constant $N_{1}$ and $N_{2}$) is

(44) $$\;đQ=dU+pdV=T^{*}dS^{*}+F_{1}df_{1}^{*}+F_{2}df_{2}^{*}.$$

We note that the net heat flow during the transition between two steady states is a combination of two exact differentials: the effective entropy $dS^{*}$ and the effective degrees of freedom $df_{1}^{*}$ (from Equation (34) it follows that $df_{2}^{*}=-\frac{x_{2}f_{1}}{x_{1}f_{2}}df_{1}^{*}$). This is contrary to the equilibrium thermodynamics, in which heat depends only on temperature and the change in entropy.

At equilibrium, the most experimentally available thermodynamic quantities are response functions such as heat capacities, compressibility, susceptibility or chemical response functions. They follow from the first law of thermodynamics and the equilibrium fundamental relationship. Using the symmetry of the second derivatives, i.e., Maxwell relations, one can express one response function in terms of others. This is very useful as, for example, the heat capacity can be determined by measurements of other quantities, such as isothermal compressibility [8].

Once we have established the fundamental relationship for steady states of binary mixtures of ideal gases, we can generalize the equilibrium response functions to steady-state response functions. First, we consider thermal response function, i.e., the heat capacity *C*, which is a measure of amount of heat needed to raise the temperature of a system by a given amount. Generally, it is defined as derivative, C=đQ/dT, but depending on which independent variables are fixed during the measurements, one has different heat capacities. For example, the heat capacity at constant volume and number of components $\left\{N_{j}\right\}$ is defined as $C_{V}=đQ/dT_{{|}_{V,\{N_{j}\}}}$, whereas $C_{p}=đQ/dT_{{|}_{p,\{N_{j}\}}}$ is the heat capacity at constant pressure and $\left\{N_{j}\right\}$. Meanwhile, in the equilibrium state at a constant volume and fixed $\left\{N_{j}\right\}$ and $\left\{f_{j}\right\}$, we can change only the temperature of the system, in the non-equilibrium steady state we have more possibilities: we can independently vary $T_{1}$ and $T_{2}$, or equivalently $T_{2}$ and the reduced difference $r=\left(T_{2}-T_{1}\right)/T_{1}$. If one changes $T_{2}$ at fixed *r* then the state parameter $f_{1}^{*}$ remains constant (see Appendix A and Equation (35)) we can define steady-state heat capacities for constant volume and pressure as follows

(45) $$C_{V}^{*}={\frac{đQ}{dT^{*}}}_{{|}_{V,\{N_{j}\}},f_{1}^{*}}$$

and

(46) $$C_{p}^{*}={\frac{đQ}{dT^{*}}}_{{|}_{p,\{N_{j}\}},f_{1}^{*}}.$$

Concerning mechanical response functions, we can generalize isothermal compressibility and thermal expansivity to the steady states as follows

(47) $$\kappa_{T,N_{j}}^{*}=-\frac{1}{V}{\left(\frac{\partial V}{\partial p}\right)}_{{|}_{T^{*},\left\{N_{j}\right\}},f_{1}^{*}}$$

and

(48) $$\alpha_{p,N_{j}}^{*}=\frac{1}{V}{\left(\frac{\partial V}{\partial T^{*}}\right)}_{{|}_{p,\{N_{j}\}},f_{1}^{*}}.$$

Because the fundamental equation of steady state has the same form as in equilibrium, the Maxwell relations for the path with r=const and $f_{1}^{*}=const$ provide connection between the thermal and mechanical response function, which is the same as in the equilibrium, i.e.,

(49) $$\kappa_{T,N_{j}}^{*}\left(C_{p}^{*}-C_{V}^{*}\right)=T^{*}V{\left(\alpha_{p,N_{j}}^{*}\right)}^{2}.$$

In order to determine $C_{p}^{*}$ one need to measure the excess heat due to the small change of $T_{2}$, which is in principle possible due to the recent development of the appropriate experimental techniques [15]. The coefficient $\kappa_{T,N_{j}}^{*}$ should be measured by changing the pressure at fixed both temperatures $T_{1}$ and $T_{2}$, while the coefficient $\alpha_{p,N_{j}}^{*}$ should be determined by varying $T_{2}$ at fixed reduced gradient *r*.

In order to control the state parameter $f_{1}^{*}$ during measurements of response function, one has to be able to determine it experimentally as a function of control parameters $T_{1},T_{2}$, and *V* for given $N_{1},N_{2}$. To this end, one needs an experimental procedure to determine $S^{*}$ and $f_{1}^{*}$ ($f_{2}^{*}$ is not an independent state parameter). Because the fundamental relation has the same form as in equilibrium thermodynamics, by performing the Legendre transform of the non-equilibrium fundamental relation, we can move to the variables $T^{*}$ and $f_{2}^{*}$. First, we measure pressure and use Equation (31) to determine $T^{*}\left(T_{1},T_{2},V\right)$. The net heat, with the use of Equations (34), (38), (40) and (44) can be written as $dQ=\alpha \left(T^{*},f_{1}^{*},V\right)dT^{*}+\beta \left(T^{*},f_{1}^{*},V\right)df_{1}^{*}$. With the non-equilibrium temperature $T^{*}$ determined as described above and the heat differential determined during the change from one to another steady state by slight change of $T_{1},T_{2}$ or *V*, we determine $f_{1}^{*}\left(T_{1},T_{2},V\right)$. This situation is similar to equilibrium thermodynamics, where mechanical and caloric measurements are necessary to determine the fundamental relation. This aspect is the same. The difference between steady-state and equilibrium thermodynamics lies in the fact that out-of-equilibrium, the measurement should be performed for a larger space of parameters. In our case, we have the two-dimensional space $T_{1}\left(=T_{2}\right),V$ in equilibrium while the three-dimensional space in steady-state $T_{1},T_{2},V$. Having the non-equilibrium parameters of state determined, $T^{*}\left(T_{1},T_{2},V\right),f_{1}^{*}\left(T_{1},T_{2},V\right),f_{2}^{*}\left(T_{1},T_{2},V\right)$ allows one to plan the experiment and change $T_{1},T_{2},V$ in such a way that $f_{1}^{*}$ is kept constant and the relation can be experimentally checked (49).

Let us consider a more general case where the system’s temperature profile is unknown. This situation appears when the heat conductivity depends on the gas density. It is straightforward to apply the integration of equations over volume and obtain the fundamental relation without any changes. As a result, the fundamental relations hold with other (not necessarily linear) temperature profiles. Moreover, the effective parameters $S^{*},f_{1}^{*},f_{2}^{*}$ can be determined experimentally according to the above-described procedure. In addition, because the pressure in the system is homogeneous, the mapping procedure also holds for the container of any shape. The introduced thermodynamic description thus works for a broad class of a binary mixture of ideal gases in vessels of different shapes and practically any temperature profile. There may be more control parameters, not only the two, that determine the temperature profile in the system. However, even when there are many control parameters, the fundamental relation with $S^{*},V,N_{1},N_{2},f_{1}^{*}$, and $f_{2}^{*}$ with only five parameters of state always carries the whole of thermodynamic information in the system and leads to thermodynamic relations such as relation (49).

## 6. Summary

The internal energy of the binary ideal gas mixture in a heat flow is the function of five parameters of state $U(S^{*},V,N_{1},N_{2},f^{*})$, irrespective of the number of boundary conditions. These parameters determine different ways of changing the internal energy of the system. The parameter $f^{*}$ is responsible for additional net heat, not included in $T^{*}dS^{*}$. $T^{*}dS^{*}$ is not the differential form describing total net heat in the system. The total net heat is given not only by the changes in $S^{*}$ but also in $f^{*}$. The same observation was made for the van der Waals gas in the heat flow. In this case, the total net heat was given not only by $S^{*}$, but also by the renormalization in the mapping procedure of two parameters of state $a^{*},b^{*}$ describing attractive interactions and the excluded volume in the van der Waals gas [16]. These parameters are constant at equilibrium since they are material parameters that define interactions in a particular system, but in non-equilibrium van der Waals gas they are parameters of state, which change the energy due to the change of density profiles. In our case of binary mixture the new parameter of state emerged from concentration profiles. Due to existence of profiles of different physical quantities, we expect all material parameters to become state parameters in the non-equilibrium systems.

Our construction of global thermodynamics for stationary states can be naturally extrapolated from binary to multi-component ideal gas mixtures in a stationary heat flow. Obviously, the number of state parameters of the stationary state will increase to include all $N_{i}$ and $f_{i}^{*}$, where i=1,…,m and *m* is the number of components. For the constant number of molecules $N=\sum_{i=1}^{m}N_{i}$, the effective parameters $f_{i}^{*}$ will not be independent. Each new parameter of state will add contribution to dU as given by Equation (41).

The formulation of the first law of global non-equilibrium thermodynamics opens the possibility to formulate the second law. The second law defines the direction of processes that take place in out-of-equilibrium systems. In our recent contributions [17] we showed that formulating the first law of stationary thermodynamics for a specific class of problems is a necessary step towards pursuing the second law. This class of problems can be extended to additional forms of energies residing in the system, e.g., Van der Waals gas (internal potential), ideal gas in gravitational field (external potential) and in this contribution, the mixture of gases (multiple components). Formulation of the second law for these system is under development.

## Data Availability

Not applicable.

## Appendix A. Variables of Steady State in Terms of Control Parameters

The integral of the concentration profile $x_{1}\left(z\right)$ in Equation (28) for the integrand given by Equation (22) with $N_{1}=N_{2}$ can be obtained analytically in terms of the Gaussian hypergeometric function ${}_{2}F_{1}\left(a,b;c;w\right)$ and polygamma function $\psi^{\left(m\right)}\left(w\right)$ [18]:

$$\begin{array}{cc} \frac{1}{L}\int_{0}^{L}x_{1}\left(z\right)dz= & \frac{1}{r\delta }\left[\psi^{\left(0\right)}\left(d\right)-\psi^{\left(0\right)}\left(d+1/2\right)\right]\\ \; & +\frac{2}{r(2+\delta )}{\left(1+r\right)}^{1+\frac{\delta }{2}}{\;}_{2}F_{1}\left(1,2d;2d+1;-{\left(1+r\right)}^{\frac{\delta }{2}}\right), \end{array}$$

where d=1/δ+1/2 with $\delta =f_{1}-f_{2}>0$ and $r=\left(T_{2}-T_{1}\right)/T_{1}>0$ is the reduced slope of the temperature profile. The state variable $f_{1}^{*}$ is thus

(A1) $$f_{1}^{*}=\frac{f_{1}}{x_{1}}\left[B_{1}\left(\delta ,r\right)+B_{2}\left(\delta ,r\right)\right],$$

where

$$\begin{array}{cc} B_{1}\left(\delta ,r\right) & =\frac{1}{r\delta }\left[\psi^{\left(0\right)}\left(d\right)-\psi^{\left(0\right)}\left(d+1/2\right)\right]\\ B_{2}\left(\delta ,r\right) & =\frac{2}{r(2+\delta )}{\left(1+r\right)}^{1+\frac{\delta }{2}}{\;}_{2}F_{1}\left(1,2d;2d+1;-{\left(1+r\right)}^{\frac{\delta }{2}}\right). \end{array}$$

For $N_{1}\neq N_{2}$ we obtain

(A2) $$f_{1}^{*}=\frac{f_{1}}{x_{1}}\left[D_{1}\left(\delta ,r,\frac{N_{1}}{N_{2}}\right)+D_{2}\left(\delta ,r,\frac{N_{1}}{N_{2}}\right)\right],$$

where

$$\begin{array}{cc} \; & D_{1}\left(\delta ,r,\frac{N_{1}}{N_{2}}\right)=-\frac{2N_{1}}{N_{2}r\left(2+\delta \right)}{\;}_{2}F_{1}\left(1,2d;2d+1;-\frac{N_{1}}{N_{2}}\right)\\ \; & D_{2}\left(\delta ,r,\frac{N_{1}}{N_{2}}\right)=\frac{2N_{1}{\left(1+r\right)}^{1+\frac{\delta }{2}}}{N_{2}r\left(2+\delta \right)}{\;}_{2}F_{1}\left(1,2d;2d+1;-\frac{N_{1}}{N_{2}}{\left(1+r\right)}^{\frac{\delta }{2}}\right). \end{array}$$

For small reduced temperature gradients across the system we find for $N_{1}\neq N_{2}$

$$\begin{array}{cc} f_{1}^{*}\approx & \;\frac{f_{1}}{x_{2}}{\;}_{2}F_{1}\left(1,2d;2d+1;-\frac{x_{1}}{x_{2}}\right)\\ \; & -\frac{f_{1}}{x_{1}}{\left(-\frac{x_{1}}{x_{2}}\right)}^{-\frac{2}{\delta }}B\left(-\frac{x_{1}}{x_{2}};2d+1;-1\right)\\ \; & +\frac{f_{1}}{x_{2}}\frac{f_{1}-f_{2}}{4{\left(1+x_{1}/x_{2}\right)}^{2}}r+O\left(r^{2}\right), \end{array}$$

where B(r;p,q) is incomplete Euler beta function [18]. For $N_{1}=N_{2}$ the above expression reduces to Equation (35).

## Appendix B. Other Approaches to Thermodiffusion

It is worth stressing that many numerical results as well as measurements of thermodiffusion coefficients are reported in the literature (see, e.g., [19,20,21,22] and references therein). They indicate that, unlike Onsager coefficients, these coefficients are functions of composition and temperature. For example, within the framework of the generalized Stefan–Maxwell thermodiffusion equations for $f_{1}=f_{2}$, one obtains exactly the same equation as Equation (23), but with $2L_{12}/\left(L_{11}k_{B}T\right)$ replaced by $\alpha_{12}=A_{12}/D_{12}$. $A_{ij}$ is the Newman–Soret thermal diffusion coefficient and $D_{ij}$ is the Stefan–Maxwell diffusivity (see e.g., Equation (6.8) from Ref. [22]). It is argued that coefficients $A_{ij}=D_{i}^{T}/\rho_{i}-D_{j}^{T}/\rho_{j}$, where $D_{i}^{T}$ is the thermal diffusivity coefficient of component *i* and $\rho_{i}$ is its mass density, are more approximately constant with respect to composition in binary systems [23]. Assuming that the ratio $\alpha_{12}$ does not depend on both temperature and composition, the solution of Equation (23) for the profile $x_{1}\left(z\right)$ is

(A3) $$x_{1}\left(z\right)=\frac{N_{1}\tilde{T}{\left(z\right)}^{\left(f_{1}-f_{2}\right)/2-\alpha_{12}}}{N_{2}+N_{1}\tilde{T}{\left(z\right)}^{\left(f_{1}-f_{2}\right)/2-\alpha_{12}}}.$$

For noble gas pairs at T=300 K, the value of $\alpha_{12}$ is equal to 0.3432 for a Ne-He pair, 0.1741 for an Ar-Ne pair, or 0.0262 for a Xe-Kr pair [24,25].
